# Factors associated with acute medication overuse in people with migraine: results from the 2017 migraine in America symptoms and treatment (MAST) study

**DOI:** 10.1186/s10194-018-0865-z

**Published:** 2018-05-24

**Authors:** Todd J. Schwedt, Aftab Alam, Michael L. Reed, Kristina M. Fanning, Sagar Munjal, Dawn C. Buse, David W. Dodick, Richard B. Lipton

**Affiliations:** 1 Mayo Clinic, 5777 East Mayo Boulevard, Phoenix, AZ 85054 USA; 2 Promius Pharma, 107 College Rd East, Princeton, NJ 08540 USA; 3 Vedanta Research, 23 Tanyard Ct, Chapel Hill, NC 27517 USA; 4 Albert Einstein College of Medicine, 1250 Waters Place, 8th Floor, Bronx, NY 10461 USA; 5 Montefiore Medical Center, The Saul R. Korey Department of Neurology, Albert Einstein College of Medicine, 1165 Morris Park Avenue, Rousso Building, Room 332, Bronx, NY 10461 USA

**Keywords:** Migraine, Medication overuse headache, Epidemiology, Adults, Allodynia

## Abstract

**Background:**

The MAST Study is a longitudinal, cross-sectional survey study of US adults with migraine. These analyses were conducted to estimate rates of acute medication overuse (AMO) and determine associations of AMO with individual and headache characteristics.

**Methods:**

Eligible respondents had ICHD-3-beta migraine, reported ≥3 monthly headache days (MHDs) in the past 3 months, ≥1 MHD in the past 30 days, and currently took acute headache medication. AMO was defined according to ICHD-3-beta thresholds for monthly days of medication taking when diagnosing medication overuse headache.

**Results:**

Eligible respondents (*N* = 13,649) had a mean age of 43.4 ± 13.6 years; most were female (72.9%) and Caucasian (81.9%). Altogether, 15.4% of respondents met criteria for AMO. Compared with those not overusing medications, respondents with AMO were significantly more likely to be taking triptans (31.3% vs 14.2%), opioids (23.8% vs 8.0%), barbiturates (7.8% vs 2.7%), and ergot alkaloids (3.1% vs 0.6%) and significantly less likely to be taking NSAIDs (63.3% vs 69.8%) (*p* < 0.001 for all comparisons). Respondents with AMO had significantly more MHDs (12.9 ± 8.6 vs 4.3 ± 4.3, *p*  <  0.001); higher migraine symptom severity (17.8 ± 2.7 vs 16.4 ± 3.0, *p*  <  0.001), higher pain intensity scores (7.4 vs 6.5, *p*  <  0.001); and higher rates of cutaneous allodynia (53.7% vs 37.5%, *p*  <  0.001). Adjusted for MHDs, the odds of AMO were increased by each additional year of age (OR 1.02, 95% CI 1.02, 1.03); being married (OR 1.19, 95% CI 1.06, 1.34); smoking (OR 1.54, 95% CI 1.31, 1.81); having psychological symptoms (OR 1.62, 95% CI 1.43, 1.83) or cutaneous allodynia (OR 1.22, 95% CI 1.08, 1.37); and greater migraine symptom severity (OR 1.06, 95% CI 1.04, 1.09) and pain intensity (OR 1.27, 95% CI 1.22, 1.32). Cutaneous allodynia increased the risk of AMO by 61% in males (OR 1.61, 95% CI 1.28, 2.03) but did not increase risk in females (OR 1.08, 95% CI 0.94, 1.25).

**Conclusions:**

AMO was present in 15% of respondents with migraine. AMO was associated with higher symptom severity scores, pain intensity, and rates of cutaneous allodynia. AMO was more likely in triptan, opioid, and barbiturate users but less likely in NSAID users. Cutaneous allodynia was associated with AMO in men but not women. This gender difference merits additional exploration.

## Background

Excessive use of acute therapies by individuals with headache has been recognized as a problem in the management of migraine for nearly 70 years [1]. According to the International Classification of Headache Disorders, Third Edition (ICHD-3), acute medication overuse (AMO) can accompany and complicate primary and secondary headaches, including migraine, tension-type headache, new daily persistent headache and posttraumatic headache, among others [2]. Although this state of using acute migraine medications too frequently is most commonly referred to as “medication overuse”, the term “acute medication overuse” is used within this manuscript since this terminology more specifically describes the condition. Overuse of drugs within certain medication classes has been associated with an increased risk of transformation from episodic to chronic migraine [3–7]. AMO is associated with greater pain intensity and disability and worse 24 h pain relief outcomes in patients with chronic migraine [8, 9], as well as the development of a secondary headache disorder known as medication-overuse headache (MOH) — at least 15 monthly headache days (MHDs) in patients with a pre-existing primary headache and developing as a consequence of regular overuse of acute headache medication for more than 3 months [2]. The term MOH implies that the overused medication is the cause of the headaches. Herein, we use AMO to describe the behavior of medication taking above a certain threshold without assumptions about causing headaches.

In the general population, about 2% of people are believed to have AMO, but headache clinics report that 50% to 70% of their patients overuse medication [5, 10–14]. Previous research has identified a number of risk factors for AMO or MOH. These include being female [15, 16], having frequent headache attacks, smoking, physical inactivity, comorbid mental health conditions and low socioeconomic status among other factors [17–24]. Many acute headache medications have been associated with AMO and MOH [2], but the highest risks are seen with barbiturate containing combination analgesics and opioids [25, 26].

Previous analyses from the 2017 MAST Study estimated that that about 35% of US adults with migraine consider their usual acute treatment to be poor or very poor (unpublished). One quarter (23.6%) never/rarely become pain-free within 2 h of taking medication, and nearly one fifth (18%) get no relief from their usual acute medication (unpublished).

The objective of the current MAST Study analysis was to estimate rates of AMO in a nonclinic sample of people with migraine and to determine associations of AMO with demographic features, migraine characteristics, and comorbidities. In prior research [9], females were at significantly greater risk than males for allodynia, and individuals with allodynia were at greater risk for AMO. Thus, we hypothesized that females would have greater odds of AMO.

## Methods

### Ethics

The information and consent form, as well as the MAST survey instrument, were reviewed by Ethical and Independent Review Services (Independence, MO), which granted an exemption under (45 CFR 46.101 [2]) and certified the exemption status of the MAST Study (#16106–01) on 31 August 2016. Before initiating the survey, respondents read a description of the study, confirmed that they understood the purpose and conduct of the study, and electronically signed informed consent to participate.

### Study design

Details about the methodology of the MAST Study have been previously published [27]. In brief, the MAST Study is a longitudinal and cross-sectional survey study of US adults 18 years or older with migraine. The baseline assessment included sociodemographics and a battery of questions about headache features, medication use, unmet treatment needs, diagnosis and consultation, and treatment response. The follow up 6- and 12-month assessments will re-evaluate these same variables in baseline cohorts to measure headache symptom and frequency changes over time.

Stratified sampling methods were used to establish a sample of respondents representative of the US adult population in sex, age, household income, race, marital status, and US Census region. Respondent demographics were maintained within 5% of 2015 US Census data.

### Recruiting and inclusion criteria

Study respondents were members of an internet research panel (Research Now, Plano, TX), which has 2.4 million active US members, that is generally representative of US demography. Panel members were invited to participate in a survey about health, and after consenting, they were asked to complete an initial screening survey that included demographics and a checklist of health conditions. Persons endorsing headache or migraine on the screening survey were evaluated with a symptom screening module that used ICHD-3 beta criteria for migraine. The symptom screening module, employed previously in the American Migraine Study (AMS) and the American Migraine Prevalence and Prevention Study (AMPP), is based on lifetime recall of symptoms associated with respondents’ most severe headaches. The AMS/AMPP module captures pain characteristics (unilateral location, pulsating/throbbing quality, moderate to severe intensity); exacerbation by routine activity; and associated symptoms (nausea, phonophobia, and photophobia), and it has a sensitivity of 100% and specificity of 82% for episodic migraine diagnosis and sensitivity of 91% and specificity of 80% for chronic migraine diagnosis [28]. Respondents meeting AMS/AMPP symptom criteria for migraine were assessed for headache frequency, and those reporting 3 or more monthly headache days (MHDs) in the past 3 months and at least 1 MHD in the past 30 days satisfied frequency criteria, completed screening, and qualified for inclusion in the study.

Only respondents currently taking medication to treat their headaches who provided self-reported monthly treatment day frequency were included in these analyses. Respondents not using medications to treat headaches and those who did not know number of days per month medication was used were excluded from the analyses.

### Assessments

The MAST Study baseline assessment used validated instruments where available. The main outcome, medication usage, was assessed by asking respondents if they were currently using prescription or nonprescription medication to treat headaches. Medications of interest for this study included simple analgesics, combination analgesics, triptans, nonsteroidal anti-inflammatory drugs (NSAIDs), barbiturates, opioids, isometheptene, and ergot alkaloids. AMO was defined according to medication use thresholds included in ICHD-3 beta diagnostic criteria for MOH [29]. AMO was considered present if a respondent reported using a triptan, opioid, barbiturate, isometheptene, ergot alkaloid medication, or combination analgesic on at least 10 days per month or an NSAID or simple analgesic on at least 15 days per month.

Covariates were obtained from single survey items and included sex (male or female); age (years); married (yes or no); education (< 4-year college degree or ≥ 4-year college degree); race (Caucasian or non-Caucasian); health insurance (yes or no); and total annual household income (<$25,000; $25,000–$49,999; $50,000–$74,999; $75,000–$99,999; and ≥ $100,000). Current tobacco use was assessed by asking respondents if they had smoked at least 100 cigarettes (lifetime recall) and if they currently smoked. Body mass index (BMI) was calculated by dividing weight in pounds by height in inches squared and multiplying by a conversion factor of 703; it is represented as a continuous variable and was used to categorize respondents as underweight (< 18.5), normal weight (18.5–24.9), overweight (25.0–29.9), and obese (≥ 30.0). Psychological symptoms (depression and/or anxiety) were based on 2-week recall and measured with the Patient Health Questionnaire for Depression and Anxiety (PHQ-4) [30].

MHD frequency was derived by asking about the number of headache days over the past 3 months (affected by headache for any part or the whole of the day), and dividing this number by 3. The MHD variable differentiated respondents using modified diagnostic criteria for episodic and chronic migraine (headache frequency of ≥ 15 MHDs for chronic and <  15 MHDs for episodic over the preceding 3 months). Chronic migraine was defined according to ICHD-3 beta criteria, but the requirement that 8 MHDs be migraine was excluded because participants would need to keep diaries and be individually interviewed, which is impractical in large population studies.

The presence of ictal cutaneous allodynia was assessed using the 12-item Allodynia Symptom Checklist (ASC-12) [31]. Migraine symptoms were measured with the Migraine Symptom Severity Scale (MSSS), a composite index that incorporates information about 7 headache features (unilateral pain, pulsatile pain, moderate or severe pain intensity, routine activities worsen pain, nausea, photophobia, phonophobia). The overall MSSS score ranges from 0 to 21 and was calculated by adding scores ranging from 0 to 3 for each of the 7 headache features assessed. Headache pain intensity was reported using a 0 to 10 scale, where 0 was no pain and 10 was the worst pain.

### Statistical analysis

Data were from the MAST baseline survey. The objective of these analyses was to provide data on respondent populations with and without AMO. Differences in sociodemographics and headache features among those with AMO versus those without AMO were of interest. Percentages were used to report dichotomous variables, including sex, marital status, education, race, health insurance status, psychological symptoms, employment, current smoking status, and cutaneous allodynia. Percentages were used to report categorical variables, including age group, BMI category, household income, and MHDs. The chi-square test was used to identify statistically significant medication overuse differences for dichotomous or categorical variables. Mean ± SD was used to report continuous variables, including age, BMI, MSSS, and pain intensity. Independent-group *t* tests were used to evaluate significant differences for continuous variables.

Six binary logistic models were run, with AMO as the outcome. The first model included the sociodemographic variables sex, age, marital status, race, household income, education, BMI, health insurance status, and smoking status. Race, household income, and health insurance status produced nonsignificant *p*-values and were excluded from subsequent analyses. In the refined models, psychological symptomology (depression and/or anxiety) and headache features (pain intensity, MSSS, cutaneous allodynia, and MHDs) were added. Additional models explored the relationship of sex and the presence of cutaneous allodynia on medication overuse. To this end, a model was run including a sex-by-allodynia interaction and 2 separate models fully adjusted for sex. Odds ratios (OR) and 95% confidence intervals (CI) are shown. *P*-values less than 0.05 were considered statistically significant, and all statistical tests were two-tailed. To control for familywise error in the multiple comparisons on sociodemographics, we applied the conservative Bonferroni adjustment (alpha 0.05/number of statistical tests). All analyses were performed in IBM SPSS Statistics, version 20.0 (IBM, Armonk, NY; 2011).

## Results

### Analysis sample

MAST Study baseline data collection started in October 2016 and ended in January 2017. Of fielded surveys, 95,821 provided usable data, 18,363 respondents met symptom criteria for migraine, and 15,133 satisfied headache frequency criteria. In total, 13,649 respondents were taking medication to treat headaches and reported monthly frequency of treatment.

### Sociodemographic characteristics

**Fig. 1:**
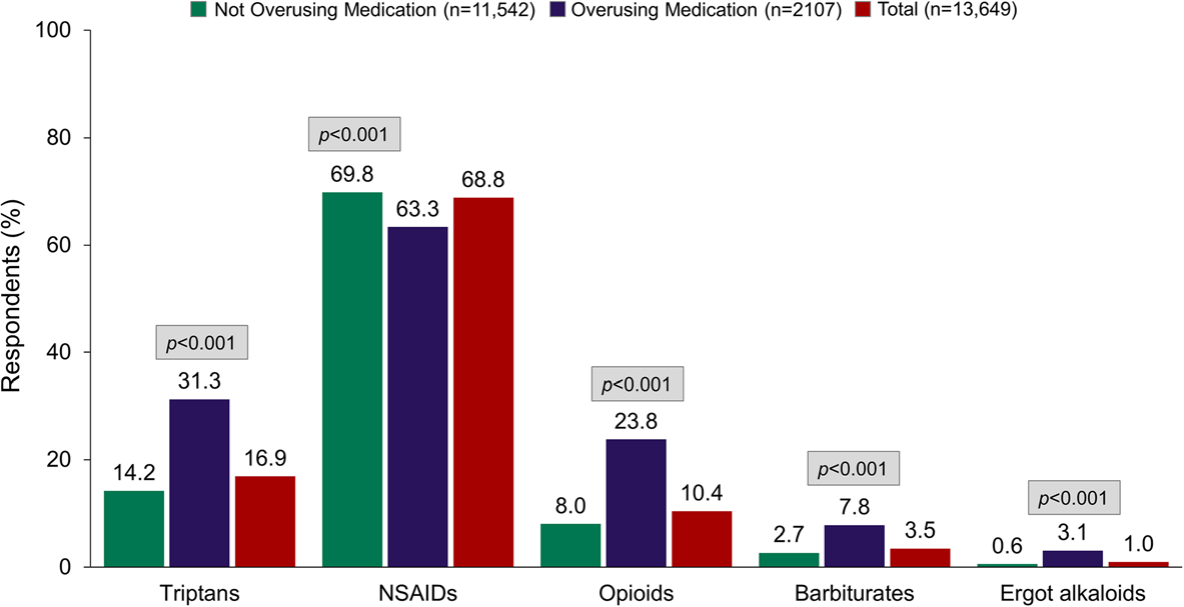
Medication Overuse by Drug Class Among Respondents with Migraine Currently Using Acute Medication for Headache. Legend: NSAID, nonsteroidal anti-inflammatory drug

The sociodemographic profile of study respondents who met study criteria is shown in Table 1. Overall, the sample had a mean age of 43.4 ± 13.6 years and was predominantly comprised of females (72.9%) and Caucasians (81.9%). AMO criteria were met by 15.4% of the population. Respondents in the AMO group were significantly more likely than those who were not overusing medications to be taking every acute headache medication class with the exception of NSAIDs. NSAID use was less likely in persons with AMO (Fig. 1, *P* < 0.001 for all comparisons). Compared with respondents who did not overuse their headache medication, those in the AMO group were significantly older (45.8 ± 13.2 vs 43.0 ± 13.6 years, *p*  <  0.001) and more likely to be male (29.5% vs 26.6%, *p*  <  0.01), had higher BMI (28.9 ± 8.5 vs 28.1 ± 7.4, *p*  <  0.001) and were more likely to be married (58.6% vs 54.3%, *p*  <  0.001). Most respondents (58.8%) had at least a 4-year college degree, but persons in the AMO group were significantly less likely than those not overusing medications to have at least a 4-year college degree (51.7% vs 60.0%, *p*  <  .001). While only 11.3% of the sample smoked cigarettes, individuals reporting AMO were significantly more likely to be smokers than those not overusing medications (18.5% vs 10.0%, *p*  <  0.001). As shown in Table 1, psychological symptoms affected 23.2% of respondents, and those in the AMO group were significantly more likely to have symptoms of depression and/or anxiety than respondents in the group not overusing medications (39.5% vs 20.2%, *p*  <  0.001).

**Table 1:** Sociodemographics of Survey Respondents with Migraine According to Medication Overuse^a^

	Not Overusing Medication *n* = 11,542	Overusing Medication *n* = 2107	Total Sample *n* = 13,649	Chi	*P*-value
Sex					
Men	3074 (26.6)	621 (29.5)	3695 (27.1)	7.136	0.008
Women	8468 (73.4)	1486 (70.5)	9954 (72.9)		
Age^b^, years (mean ± SD)	43.0 ± 13.6	45.8 ± 13.2	43.41 ± 13.62	8.891	< 0.001
Age group, years					
18–24	899 (7.8)	80 (3.8)	979 (7.2)	96.346	< 0.001
25–34	2831 (24.5)	409 (19.4)	3240 (23.7)		
35–44	2775 (24.0)	518 (24.6)	3293 (24.1)		
45–54	2566 (22.2)	546 (25.9)	3112 (22.8)		
55–64	1572 (13.6)	348 (16.5)	1920 (14.1)		
65–74	815 (7.1)	184 (8.7)	999 (7.3)		
75+	84 (0.7)	22 (1.0)	106 (0.8)		
Married					
No	5261 (45.7)	867 (41.4)	6128 (45.1)	13.436	< 0.001
Yes	6245 (54.3)	1229 (58.6)	7474 (54.9)		
Race					
Non-Caucasian	2091 (18.2)	361 (17.2)	2452 (18.1)	1.166	0.280
Caucasian	9379 (81.8)	1736 (82.8)	11,115 (81.9)		
Household income, $					
< 25,000	1254 (11.2)	292 (14.2)	1546 (11.6)	17.025	0.002
25,000–49,999	2422 (21.6)	444 (21.6)	2866 (21.6)		
50,000–74,999	2493 (22.2)	437 (21.3)	2930 (22.1)		
75,000–99,999	1986 (17.7)	363 (17.7)	2349 (17.7)		
≥ 100,000	3070 (27.3)	516 (25.1)	3586 (27.0)		
Education, ≥4-year college degree					
No	4612 (40.0)	1018 (48.3)	5630 (41.2)	51.000	< 0.001
Yes	6930 (60.0)	1089 (51.7)	8019 (58.8)		
BMI^b^, kg/m^2^ (mean ± SD)	28.1 ± 7.4	28.9 ± 8.5	28.3 ± 7.6	4.380	< 0.001
BMI category					
Underweight	329 (2.9)	94 (4.5)	423 (3.1)	44.240	< 0.001
Normal	4154 (36.0)	645 (30.6)	4799 (35.2)		
Overweight	3387 (29.3)	588 (27.9)	3975 (29.1)		
Obese	3672 (31.8)	780 (37.0)	4452 (32.6)		
Health insurance					
No	945 (8.2)	184 (8.7)	1129 (8.3)	0.628	0.428
Yes	10,597 (91.8)	1923 (91.3)	12,520 (91.7)		
Psychological symptoms^c^					
No	9210 (79.8)	1275 (60.5)	10,485 (76.8)	370.957	< 0.001
Yes	2332 (20.2)	832 (39.5)	3164 (23.2)		
Current smoker					
No	10,387 (90.0)	1718 (81.5)	12,105 (88.7)	126.127	< 0.001
Yes	1155 (10.0)	389 (18.5)	1544 (11.3)		

*SD* standard deviation, *BMI* body mass index

^a^Values are n (%) unless otherwise indicated

^b^Determined by *t* test

^c^Anxiety and/or depression, as determined by the Patient Health Questionnaire for Depression and Anxiety (PHQ-4)

### Headache features

Table 2 shows that respondents in the AMO group reported significantly more MHDs than those not overusing medications (12.9 ± 8.6 vs 4.3 ± 4.3, *p*  <  0.001). The mean MSSS score of the total sample was 16.6 ± 3.0, and MSSS scores were significantly higher among those in the AMO group than in those not overusing medications (17.8 ± 2.7 vs 16.4 ± 3.0, *p*  <  0.001). Allodynia was present in 40.0% of the total sample, and those in the AMO group were significantly more likely to be allodynic than individuals not overusing medications (53.7% vs 37.5%, *p*  <  0.001). Pain intensity scores were significantly higher in the AMO group than in those not overusing medications (7.4 vs 6.5, *p*  <  0.001), as shown in Table 2. After application of the Bonferroni adjustment, AMO differences on each of the 11 sociodemographic parameters and the 4 headache features remained statistically significant (*p* < 0.003 for all parameters).

**Table 2:** Headache Features and Characteristics of Respondents with Migraine According to Medication Overuse

	Not Overusing Medication *n* = 11,542	Overusing Medication *n* = 2107	Total Sample *n* = 13,649	Chi	*P*-value
MHDs^a^ (mean ± SD)	4.3 ± 4.3	12.9 ± 8.6	5.6 ± 6.0	69.7	< 0.001
MHD category, n (%)					
0–4	8054 (69.8)	451 (21.4)	8505 (62.3)	2830.498	< 0.001
5–9	2351 (20.4)	380 (18.0)	2731 (20.0)		
10–14	656 (5.7)	417 (19.8)	1073 (7.9)		
≥ 15	481 (4.2)	859 (40.8)	1340 (9.8)		
Cutaneous allodynia, n (%)					
No	7208 (62.5)	976 (46.3)	8184 (60.0)	192.380	< 0.001
Yes	4334 (37.5)	1131 (53.7)	5465 (40.0)		
MSSS^a^ (scale of 0–21)	16.4 ± 3.0	17.8 ± 2.7	16.6 ± 3.0	19.433	< 0.001
Pain intensity^a^ (scale of 0–10)	6.5 ± 1.6	7.4 ± 1.6	6.7 ± 1.6	22.7	< 0.001

*MHD* monthly headache day, *SD* standard deviation, *MSSS* Migraine Symptom Severity Score

^a^Determined by independent-group *t* test

### Logistic regression

Covariates were entered sequentially to an initial model predicting AMO as a dichotomous outcome. The initial sociodemographic model (Table 3, column 1) showed that AMO was associated with increases in age and BMI, being married, and currently smoking, whereas having at least a 4-year college degree was protective of AMO. Race, household income, and having health insurance did not significantly contribute and were trimmed from subsequent analyses. Respondents’ sex, while not significantly associated with AMO, was included in all models.

**Table 3:** Risk (odds ratios [95% CI]) of Medication Overuse Among Respondents with Migraine Currently Using Acute Medication for Headache (N = 13,649)

	Model					
	1	2	3	4	5	6
	Sociodemographics	Psychological symptoms^a^	Headache characteristics	Sex-by-allodynia interaction	Men	Women
Male	1.05 (0.95, 1.17)	1.01 (0.91, 1.13)	1.32 (1.16, 1.50)	1.10 (0.93, 1.31)	–	–
Age	1.01 (1.01, 1.02)	1.02 (1.01, 1.02)	1.02 (1.02, 1.03)	1.02 (1.02, 1.03)	1.02 (1.01, 1.03)	1.02 (1.02, 1.03)
Married	1.12 (1.02, 1.24)	1.19 (1.07, 1.31)	1.19 (1.06, 1.34)	1.18 (1.05, 1.33)	1.44 (1.14, 1.83)	1.10 (0.95, 1.26)
< 4-year college degree	0.80 (0.72, 0.88)	0.85 (0.77, 0.94)	0.99 (0.88, 1.12)	0.99 (0.88, 1.11)	1.13 (0.90, 1.41)	0.93 (0.81, 1.07)
BMI	1.01 (1.01, 1.02)	1.01 (1.00, 1.01)	1.00 (1.00, 1.01)	1.00 (1.00, 1.01)	1.44 (1.14, 1.83)	1.00 (1.00, 1.01)
Psychological symptoms^a^	–	2.61 (2.36, 2.89)	1.62 (1.43, 1.83)	1.60 (1.41, 1.81)	1.94 (1.54, 2.45)	1.48 (1.28, 1.72)
Current smoker	2.05 (1.80, 2.33)	1.78 (1.56, 2.03)	1.54 (1.31, 1.81)	1.52 (1.30, 1.79)	1.65 (1.25, 2.16)	1.42 (1.16, 1.74)
Cutaneous allodynia	–	–	1.22 (1.08, 1.37)	1.09 (0.95, 1.25)	1.61 (1.28, 2.03)	1.08 (0.94, 1.25)
MHDs (vs 0–4, reference)			1.0			
5–9	–	–	2.50 (2.15, 2.90)	2.50 (2.15, 2.90)	2.46 (1.89, 3.20)	2.52 (2.10, 3.01)
10–14	–	–	9.73 (8.27, 11.45)	9.75 (8.28, 11.47)	8.20 (6.03, 11.16)	10.44 (8.60, 12.67)
≥ 15	–	–	27.34 (23.43, 31.89)	27.45 (23.53, 32.04)	21.01 (15.64, 28.21)	30.25 (25.20, 36.30)
MSSS	–	–	1.06 (1.04, 1.09)	1.06 (1.04, 1.09)	1.06 (1.02, 1.10)	1.06 (1.03, 1.09)
Pain intensity	–	–	1.27 (1.22, 1.32)	1.27 (1.22, 1.32)	1.21 (1.13, 1.31)	1.28 (1.22, 1.35)
Sex-by-allodynia interaction	–	–	–	1.53 (1.18, 1.97)	–	–

*MHD* monthly headache day, *MSSS* Migraine Symptom Severity Scale

^a^Anxiety and/or depression, as determined by the Patient Health Questionnaire for Depression and Anxiety (PHQ-4)

Adding psychological symptoms from the PHQ-4 to the adjusted sociodemographic model (Table 3, column 2) showed that the presence of depression and/or anxiety increased the odds of AMO by 2.6 times relative to those without psychological symptoms. In the fully adjusted model — sociodemographics plus psychological symptoms and headache features (Table 3, column 3) — the risk of AMO was increased versus the lowest MHD category (0 to 4) among those with 5 to 9 MHDs, 10 to 14 MHDs, and at least 15 MHDs. After adjusting for MHDs, the odds of being in the AMO group were increased by additional years of age, marriage, smoking, psychological symptoms, cutaneous allodynia, additional MSSS points, and greater pain intensity. Education and BMI were not associated with AMO in the fully adjusted model.

Although we predicted that females would have greater odds of AMO, the fully adjusted model revealed that relative to women, males had an increased likelihood of being in the AMO group (OR 1.32, CI 1.16, 1.50). To explore this unexpected finding, a final model included a sex-by-allodynia interaction (Table 3, column 4). Because the interaction was significant (OR 1.53, 95% CI 1.18, 1.97), we ran separate models for men and women to explore the relationship more completely. As shown in Table 3, columns 5 and 6, men with cutaneous allodynia were at higher odds for the development of AMO (61%) compared to women with cutaneous allodynia (8%).

## Discussion

This analysis of MAST Study data was conducted to estimate rates of AMO in a representative sample of people with migraine and to determine associations of AMO with individual and migraine characteristics. Overall, 15.4% of MAST respondents met criteria for AMO. Compared with respondents who were not overusing acute medications, those who were overusing acute medications were less likely to be using NSAIDs, but they were more than twice as likely to be using triptans and nearly 3 times as likely to be using barbiturates or opioids. Though persons with AMO were 5 times more likely to be using ergot alkaloids, ergot use was rare. The likelihood of AMO was significantly increased among respondents who were male, older, married, less educated, smokers, and in a higher BMI category, as well as those who had symptoms of anxiety or depression, cutaneous allodynia, greater severity of migraine symptoms, and greater pain intensity. Race, household income, and health insurance status had no effect on the incidence of AMO. Although AMO should be discussed with all patients who have frequent headaches who use acute medications, identification of factors that are associated with AMO can help clinicians determine with which patients the risk of AMO should be an area of greater focus. Furthermore, although cause-and-effect relationships cannot be determined from this cross-sectional analysis, it is possible that intervening on certain factors, like anxiety and severe migraine attack symptoms, could reduce the likelihood of developing AMO.

Some of the most important MAST Study findings align with earlier work. The overall rate of AMO in the MAST Study is similar to the results of previous studies reporting that 17% to 18% of adults with migraine met criteria for AMO [32, 33]. The results for individual agents are also compatible with prior research showing that migraine progression and AMO are positively associated with the use of triptans, opioids, and ergot alkaloids [5, 7, 26] and negatively associated with NSAIDs [26]. The finding that AMO was more likely among MAST Study respondents in higher BMI categories and who currently smoke confirms previous research showing a higher prevalence of MOH among individuals with a BMI of at least 30 [16] and smokers [34].

Other MAST Study results differ from those previously reported. For example, the rate of allodynia in the total sample (~ 40.0%) is similar to some previous studies [35] but lower than the 50% to 80% found in other studies [31, 36–41]. We are not sure what accounts for the variation in rates of allodynia among studies using the ASC-12. However, the greater risk for AMO among men with allodynia versus women with allodynia observed in this study is a new finding. The odds of being in the AMO group were elevated among men, and the presence of allodynia increased the likelihood of AMO in men by more than 60%. Reasons for the sex differences in the frequency of AMO and in the association of allodynia with AMO are unknown. Possibilities include: 1) biologic differences in the way that men and women experience allodynia; 2) variations in how men and women report symptoms of allodynia, including how they respond to questions on the ASC-12; 3) differential effects of having allodynia on the decision to take acute migraine medications; and 4) differential effects of AMO on the development of allodynia. Future studies are needed to test these different theories.

We expected that AMO would be associated with attack frequency in MAST Study respondents. However, after adjusting for MHDs, pain intensity, and sociodemographics, those with AMO were still significantly more likely to have symptoms of depression and/or anxiety than respondents who were not overusing medications, and individuals with psychological symptoms were almost 3 times as likely to be overusing medication as those who were not overusing medication. Future studies are needed to determine if reductions in anxiety and/or depression correlate with reductions in the frequency of taking acute migraine medications.

Strengths of the study include its use of a large and well-defined sample that was generally representative of current US demography, and the inclusion of validated assessments of symptoms of other relevant respondent characteristics. Limitations of the study include the inability of cross-sectional population-based study to record data that would establish a causal relationship between AMO and increasing MHDs [2]. We also did not assess risk of AMO by individual medication class (ie, MAST drug class usage categories are not mutually exclusive), information that might have helped to guide drug choices and targeted educational efforts in clinical practice.

## Conclusions

Approximately 15% of persons with migraine met criteria for AMO. As expected, people with frequent attacks were more likely to have AMO than those with less frequent attacks. After adjusting for headache frequency, the odds of being in the AMO group increased with each additional year of age, each 1-point increase in BMI, being married, smoking, psychological symptoms, cutaneous allodynia, each additional MSSS point, and with greater pain intensity. Of note, males with allodynia were more likely to meet criteria for AMO than females with allodynia. Although this cross-sectional study cannot determine temporal sequence or causality for these associations, treating modifiable predictors of AMO is good clinical practice.

## Abbreviations

AMPP: American Migraine Prevalence and Prevention Study
AMS: American Migraine Study
ASC-12: Allodynia Symptom Checklist
ICHD: International Classification of Headache Disorders
MAST: Migraine in America Symptoms and Treatment
MHD: Monthly headache day
MIDAS: Migraine Disability Assessment Scale
MSSS: Migraine Symptom Severity Score
NSAID: Nonsteroidal anti-inflammatory drug
OTC: Over-the-counter
PHQ-4: Patient Health Questionnaire for Depression and Anxiety
SD: Standard deviation
